# A Case-based Approach to Pacemakers, ICDs and Cardiac Resynchronisation: Questions for Examination Review and Clinical Practice. Editors: Paul A. Friedman, Melissa A. Rott, Anita Wokhlu, Samuel J. Asirvatham, David L. Hayes. Cardiotext Publishing, LLC

**Published:** 2012-04-30

**Authors:** Narayanan Namboodiri

**Affiliations:** Sree Chitra Tirunal Institute for Medical Sciences and Technology, Trivandrum, Kerala, India

Optimal management of patients with implantable cardiac devices has become crucial, in the rapidly evolving field of cardiac electrophysiology. A clear understanding of device algorithms and growing programming options is essential to decipher many complex device behaviours. As any other areas in cardiac electrophysiology, working through unknown tracings as examples is the best way to familiarise with them.

Written by experts from Mayo Clinic, Rochester, Minnesota, - Paul A. Friedman, Melissa A. Rott, Anita Wokhlu, Samuel J. Asirvatham, and David L. Hayes - *"A case-based approach to pacemakers, ICDs and cardiac resynchronisation: questions for examination review and clinical practice"* provides electrophysiologists, fellows-in-training, nurses, and cardiovascular technicians involved in day-to-day management of device patients with detailed information about many device behaviour algorithms. This outstanding work has come in 2 volumes of case studies that encompass variations of normal and abnormal function of pacemakers, ICDs, and CRT devices.

The first volume of this work includes introductory and intermediate cases, whereas the second volume contains additional intermediate cases as well as advanced cases. Both volumes provide a wealth of clinically relevant material and case studies which help the reader to achieve the goal of advancing general concepts in device management. The case studies are discussed by five clinicians with differing backgrounds in a simple way so as to make them applicable to a variety of caregivers. The major stress has been given to discuss the tracings with their clinical relevance and their usefulness to illustrating general principles, practical tips, or interesting findings in device practice.

To me, this book appears to serve two basic purposes to the reader: First, it provides a self-evaluation of practical comprehension of cardiac device function and analysis. There are nearly 100 case studies with questions, answers, discussion and references to provide an overview of what is frequently encountered in the clinical arena of these disciplines. The second purpose is that it provides a practical and educational guide to assist in conducting effective device follow-up. The case scenarios discussed in this book are particularly useful in device follow-up clinics and training programs. Furthermore, the material is designed to be helpful in pinpointing strengths and weaknesses in the user's grasp of the material and to provide a realistic overview of typical clinical experience.

Clinical problems being encountered in practice are largely unique as they are patient-specific. Realising this important fact, the authors have made every effort to present them in a systematic way almost similar to what is being observed in a real-world scenario. A case history is followed by relevant images and a multiple choice question. This helps the reader to solve these problems by carefully analysing them to reach at the most likely possibility. The answer with a detailed explanation is given in the subsequent pages. The cases generally progress from simpler to more complex cases. An appendix is provided that identifies the major diagnostic dilemma presented by each case, and the index will direct the reader to cases and discussions focussing on specific issues. No doubt, these cases are well-suited for a clinician who does regular follow up of the patients, or who takes an examination related to device management.

